# Camouflage Using Surface Disruption: The Importance of Corners Versus Edges

**DOI:** 10.1002/ece3.72052

**Published:** 2025-08-21

**Authors:** Ruby McLellan, Vanessa K. Bowden, Troy A. W. Visser, Jennifer L. Kelley

**Affiliations:** ^1^ School of Biological Sciences The University of Western Australia Perth Western Australia Australia; ^2^ School of Psychological Science The University of Western Australia Perth Western Australia Australia

**Keywords:** background matching, depth perception, disruptive colouration, shape recognition, shape‐from‐shading, visual perception

## Abstract

Disruptive colouration is a common mode of camouflage used by predators and prey to conceal their body contours. However, it is unclear how disruptive colouration hinders the detection and recognition of three‐dimensional (3D) body shapes. In human visual systems, corners, not edges, of an object are critical for shape processing and contour completion. However, whether corners are also critical for 3D shape recognition in non‐human animals has not been investigated. To test this, in Experiment 1 we presented 3D‐printed moth‐like targets with either corner disruption, edge disruption or no disruption to wild, free‐living birds. We repeated this in Experiment 2 with 2D targets, comprising images matched to Experiment 1, to determine if pictorial cues (patterns that might produce an impression of depth) had the same effect. Contrary to our predictions, we found no effect of surface disruption location on survival probability for either 3D or 2D moth targets. In contrast to previous work, we found that targets with disrupted surfaces, irrespective of the disruption location, did not have higher survival than those with continuous surfaces. All targets were designed to match the background, so perhaps there were no further camouflage benefits of surface disruption. However, when the data from both experiments were combined, we found that 3D targets had higher survival than 2D targets, perhaps because moths are typically flattened, so this shape is less familiar to the birds. Most of the variation in our data was explained by the spatial and temporal structure of the data, owing to different predator communities and seasonal variation in predation risk. Given corners are critical for shape recognition in humans, developing an appropriate system to test visual perception in non‐human animals will provide key insights into the role of visual perception in the predator–prey arms race.

## Graphical abstract

Disruptive colouration allows animals to avoid predation by concealing the body's outline or surfaces. If corners are important for shape processing, as in human vision, then corner disruption should provide better camouflage than edge disruption. Although the location of surface disruption did not influence survival, we found that 3D prey had higher survival than 2D prey, suggesting predator preference for prey body shape.

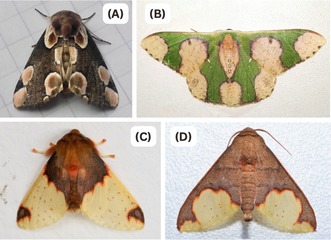

## 1 Introduction

Camouflage in the animal kingdom functions as a form of concealment and is often an adaptive response to the cognitive and perceptual processes of predators (Merilaita et al. 2017; Skelhorn and Rowe 2016; Troscianko et al. 2009). While there are a number of different ways of achieving camouflage, one that is particularly intriguing is disruptive colouration, which is a set of markings that functions to conceal the body's contours by breaking up the body's outline and/or shape surfaces (Cuthill et al. 2005; Stevens and Merilaita 2009). Numerous examples of disruptive colouration can be observed in nature, in animals such as the common cuttlefish ( *Sepia officinalis* ) (Kelman et al. 2007), the spotted marsh frog ( *Limnodynastes tasmaniensis* ) (Osorio and Srinivasan 1991), the marine isopod (*Idotea baltica*) (Merilaita 1998) and the common shore crab (*Carcinus maenus*) (Price et al. 2019). Disruptive colouration is thought to disrupt predators' visual processes; internal patterns may enhance noise and provide misleading cues by creating prominent false edges that distract from the animal's true shape (Cuthill 2019; Merilaita et al. 2017; Osorio and Srinivasan 1991).

There is good evidence that disruptive colouration is a successful strategy for avoiding predation (Cuthill et al. 2005; Fraser et al. 2007; Hanlon et al. 2008; Merilaita and Lind 2005; Price et al. 2019; Schaefer and Stobbe 2006). For example, field experiments using wild, free‐living birds as predators have found that artificial, two‐dimensional (2D) moths with high‐contrast edge patterns have higher survivability than non‐disrupted targets (Cuthill et al. 2005; Fraser et al. 2007; Schaefer and Stobbe 2006). Studies using humans as predators and computer‐generated moth images have confirmed these field results, showing that disruptive markings that intersect with the object's boundaries provide optimal camouflage (Fraser et al. 2007; Stevens and Cuthill 2006; Webster et al. 2013). Studies of prey behaviour also reveal the importance of disruptive colouration for camouflage; moths choose backgrounds that maximise the disruptive effect produced by their wing patterns (Kang et al. 2015), while cuttlefish produce disruptive patterns when the background is dark but contains large, light‐coloured objects (Kelman et al. 2008). Collectively, these studies reveal the importance of disruptive colouration as a camouflage strategy in nature.

Disruptive colouration is thought to be effective because it may exploit several low‐level visual processing mechanisms. This is particularly the case for high‐contrast elements, such as enhanced edges, where light colour patches are bordered by lighter edges and dark patches have darker borders. For example, Osorio and Srinivasan (1991) showed that the response of edge detectors to enhanced edges markings in the spotted marsh frog ( *L. tasmaniensis* ) is indistinguishable to that elicited by the frog's natural body outline. Enhanced edges may also produce a false impression of depth, providing camouflage by interfering with the Gestalt ‘principles of grouping’ (Kelley et al. 2022; Merilaita et al. 2017; Osorio and Cuthill 2014), where the human brain groups basic visual stimuli into coherent, recognisable objects or scenes (Wolfe et al. 2021). For example, studies with humans have found that snake‐like targets with edge enhancement are difficult to detect because these markings produce the impression of depth which may be similar to that found in the background (Adams et al. 2019; Egan et al. 2016). Targets with edge enhancement are also slower to identify than targets without depth cues, suggesting that surface continuity is important for object identification (Sharman et al. 2018).

Much of our understanding of object detection and recognition comes from studies of human vision. In humans, the early stages of visual processing are strongly influenced by the physical geometry of the stimulus (Wolfe et al. 1992). Attneave (1954) demonstrated that the information most relevant for object recognition is concentrated at points where boundary curvature is at its maximum—that is to say, at the corners of the object. More recent work (Bowden et al. 2015; Dickinson et al. 2018; Poirier and Wilson 2007) supports this, showing that objects are identified by the shape and positioning of their corners, which assist in shape perception more than the sections of contour joining the corners (referred to here as ‘edges’). Further, in a visual search task, shapes with corners were more easily detected amongst ellipses (i.e., shapes without corners) than vice versa (Kristjánsson and Tse 2001). These results suggest that abrupt changes in an object's boundary can be quickly detected and used to extract essential information about the nature of an object.

According to the ‘global feature disruption hypothesis’ (Webster 2015), any disruption of shape processing should impair object identification. However, if boundary curvature is a key factor in object identification, as suggested by human vision studies (Bowden et al. 2015; Dickinson et al. 2018; Poirier and Wilson 2007), then an optimal camouflage strategy should be to disrupt edge detection on boundaries where maximum curvature occurs, in other words, to obscure the corners. This should be superior to edge disruption at other, non‐corner locations on the boundary of an object.

An additional rationale for our study stems from the fact that camouflage using disruptive colouration works by preventing recognition of an animal's outline or shape (Stevens and Merilaita 2011); yet almost all experimental studies to date have focused on two‐dimensional prey targets and their outlines (e.g., Cuthill et al. 2005; Fraser et al. 2007; Stevens and Cuthill 2006). This has left a gap in our understanding about how camouflage works when considering three‐dimensional body shapes and variations in depth across the body's surfaces. To emphasise the 3D component of disruptive colouration, we hereafter use the term ‘surface disruption’ whereby the object's surface is not continuous but is, or appears to be, broken into sections of varying depth (note that this terminology differs from that used by Stevens et al. 2009). We use the term ‘pictorial cues’ to mean patterns that could produce the same effect by producing a false impression of depth (Cott 1940; Kelley et al. 2022). Here, we use both 3D and 2D prey shapes to determine whether the location of disruption is important for camouflage. To our knowledge, this represents a novel application of human vision research to the study of animal camouflage mechanisms.

**FIGURE 1:**
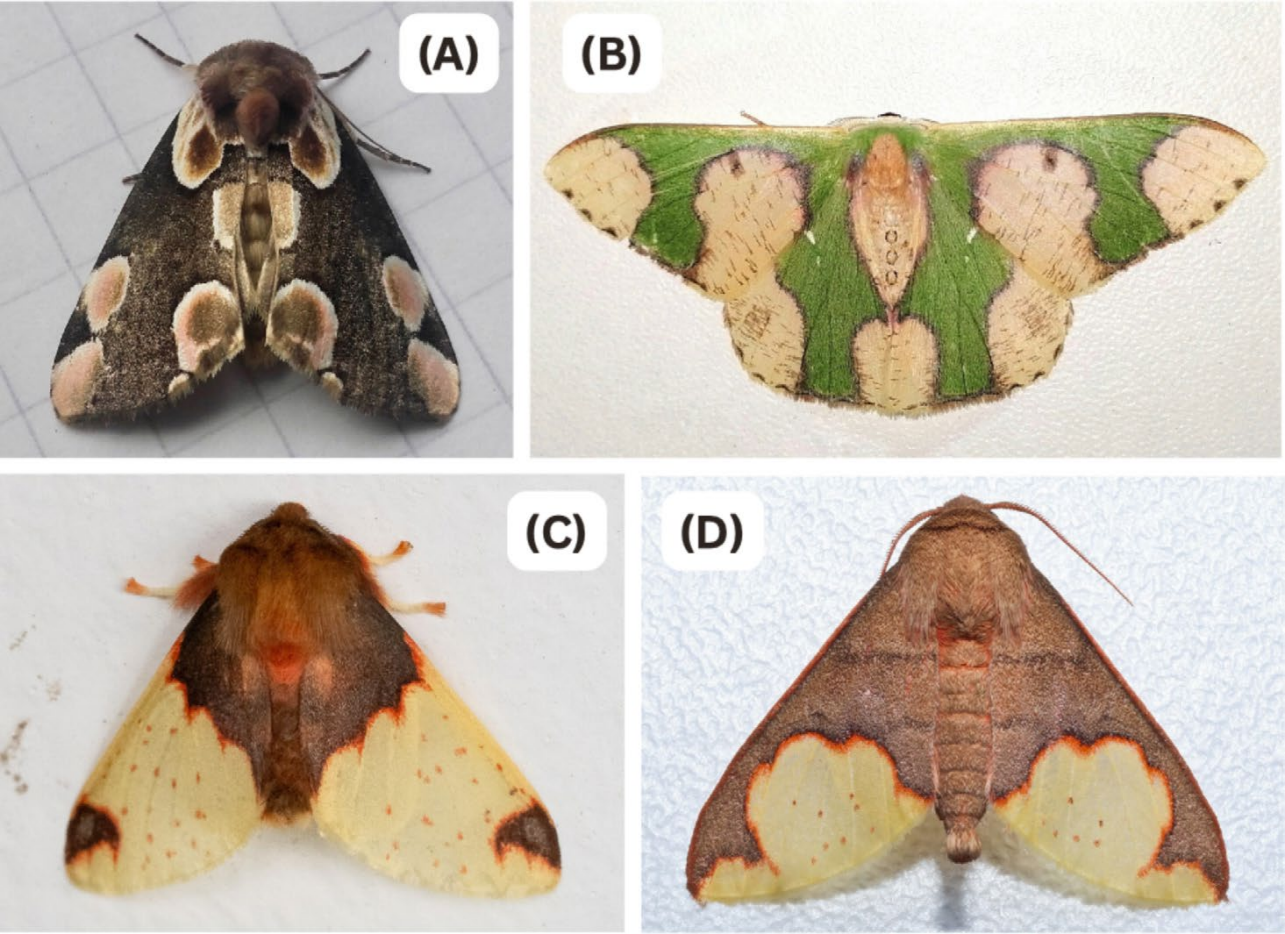
Four examples of edge and corner marginal markings found in nocturnal lepidopteran wings. (A) *Thyatira batis (Image credit:* Chrisdo44229, 2023, iNaturalist: https://www.inaturalist.org/observations/183849077), (B) *Oospila* *albicoma* (Image credit: Gundogdu, F., 2020, iNaturalist: https://www.inaturalist.org/observations/57080633), (C) *Pseudepimolis haemasticta* (*Image credit: Öberg, E., 2024*, iNaturalist: https://guatemala.inaturalist.org/observations/227368433), (D) *Pseudepimolis incisa* (Image credit: Schulz, E., 2021, iNaturalist: https://guatemala.inaturalist.org/observations/97001105).

To test this hypothesis, this study aimed to determine whether the location of surface disruption on a 3D shape influences the survival probability of moth‐like targets presented to wild, free‐living bird predators. Following previous work on surface disruption using 3D targets (King et al. 2023), we also tested 2D targets with equivalent pictorial cues (patterns that may produce an impression of depth) to determine if these could produce a similar effect. We designed our artificial prey based on moths because they display disruptive patterns, including those that may provide strong edge cues by inducing an impression of depth (pictorial cues) (Egan et al. 2016; Kelley et al. 2023; Kelley et al. 2022), and because these markings are observed at different positions on the wing surface (Figure 1). In Experiment 1, the targets were all 3D‐printed shapes with varying locations of surface disruption. In Experiment 2, the targets were 2D paper constructions that used pictorial cues to create the same surface disruption conditions as Experiment 1. Experiment 2 therefore allowed us to determine whether pictorial cues could potentially provide the same survival benefits as physical surface disruption. In both experiments, targets had either corner disruption, edge disruption or no disruption (i.e., continuous flat surface).

If surface disruption is an effective camouflage strategy, as shown previously (Costello et al. 2020; King et al. 2023), then both 3D and 2D targets with continuous complete surfaces should have lower survival than those with disrupted surfaces. In Experiment 1, we expected that 3D targets with corner disruption should be harder to recognise and hence should have higher survival than 3D targets with edge disruption. If pictorial cues can produce a false impression of depth, then the findings should be the same for 2D targets in Experiment 2.

## 2 Methods

### 2.1 Experiment 1: 3D Target Design

**FIGURE 2:**
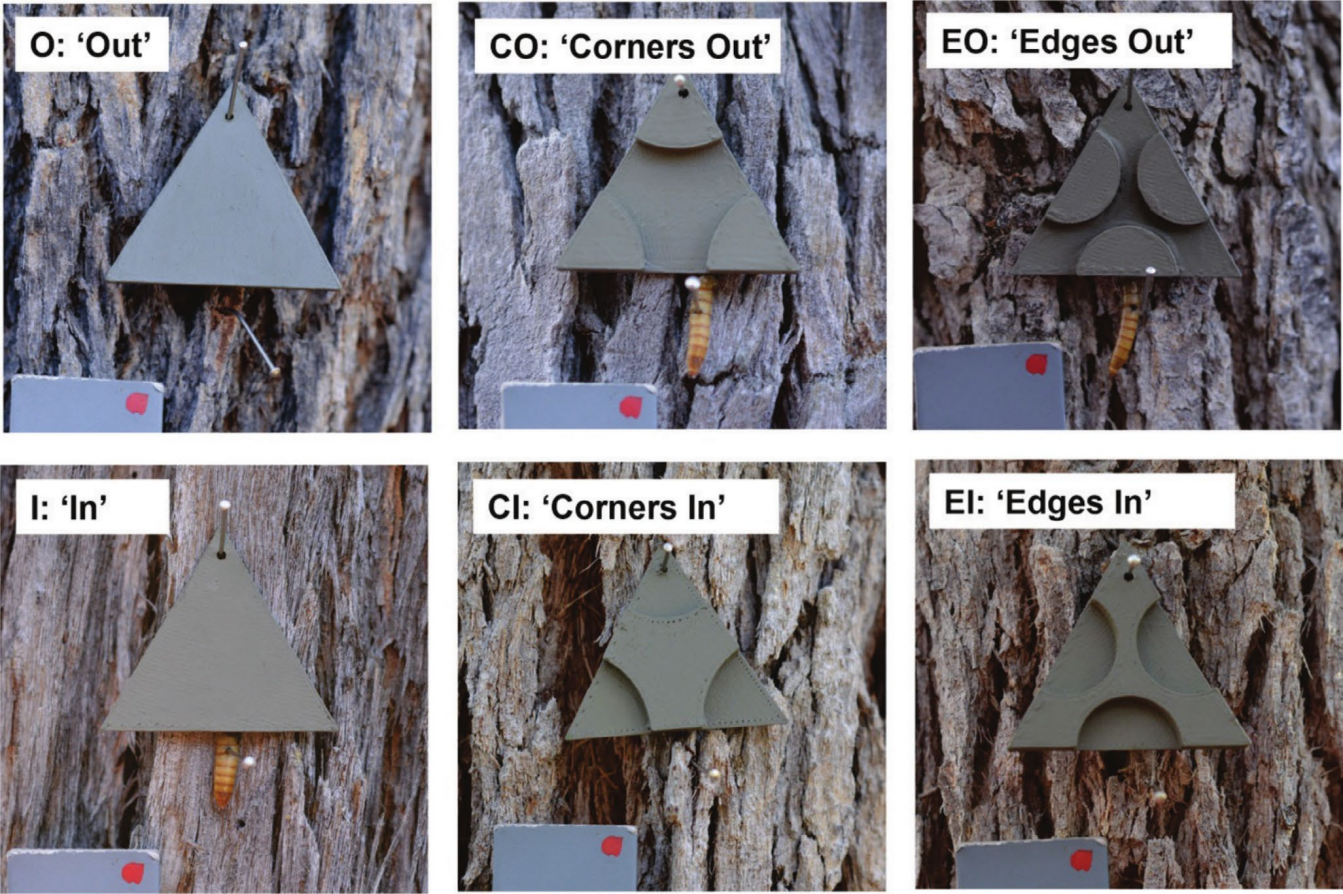
3D‐printed artificial ‘moth’ targets and their associated names, including surface disrupted targets (CI, corners in; CO, corners outs; EI, edges in; EO, edges out) and continuous surface targets (I: in, O: out). ‘I’ refers to 1 mm width and ‘O’ to 5 mm target width. Targets O, CI and EI have undergone predation (mealworm absent) while targets CO, EO and I have not (mealworm present). Images were captured between 7 am and 8 am on the 3rd April 2024 with an 18% grey standard included in each photo (bottom left). All targets shown here are pinned on marri bark ( *Corymbia calophylla* ), except for I which is on jarrah bark ( *Eucalyptus marginata* ).

The targets were designed using Blender 3D modelling software and printed with a Pruser 3D printer (Original Prusa i3 MK3S+) using PLA filament. Targets were equilateral triangles with sides of 4 cm in length and 3.464 cm in height (Figure S1). An equilateral shape allowed us to control for the spatial proximity of corners and was appropriate given moths are roughly triangular shaped in a resting position (Figure 1) and many prior camouflage studies using wild birds have used triangular targets (e.g., Cuthill et al. 2005; Fraser et al. 2007). We created six 3D‐printed targets that either exhibited no surface disruption or surface disruption of edges and corners (Figure 2). Here, when referring to the target treatments, we use the term ‘boundary’ to mean the entire periphery of the object and ‘edge’ to refer to the segments of the boundary between the corners. The boundary of the object (i.e., prey target) is therefore comprised of edges and corners.

Disruption of a 3D surface, or the same effect using the equivalent pictorial cues, can produce a shape where the corners or edges may be seen (or perceived) as protruding or cut away (King et al. 2023). Based on this, each treatment, with different parts of the surface protruding (‘out’) or cut away (‘in’), was assigned a corresponding code name, (see Figure 2; Corners In = CI, Corners Out = CO, Edges In = EI, Edges Out = EO). There were two control treatments with no disruption, one with the whole surface cut away (‘in’ = I; 1 mm thick) and one with the whole surface protruding (‘out’ = O; 5 mm thick). These differences in thickness between the controls represent the two different surface heights present in the treatments with disrupted surfaces: the protruding surfaces were 5 mm thick and the cut away surfaces were 1 mm thick. Surface sectors that were ‘in’ had 1 mm thickness and those that were ‘out’ had 5 mm thickness. Each edge and corner sector, in the surface disrupted targets, had an area of 1.155 cm^2^ (Table S1). The collective surface area of the three sector components was equal to the remaining surface area of the triangle, 3.464 cm^2^ (i.e., the area ‘in’ was equal to the area ‘out’; Table S1). We expected that the ‘Corners In (CI)’ and ‘Edges Out (EO)’ treatments would have the highest survival of the surface disrupted treatments, as their inward corners and protruding edges could interfere with boundary resolution and impede object detection (Webster 2015). In contrast, we expected the ‘Edges In (EI)’ and ‘Corners Out (CO)’ targets to have the lowest survival, because the corners were present in both these treatments and offer crucial information regarding the object's underlying structure and shape (Bowden et al. 2015) which should facilitate object recognition.

Our targets were not designed to imitate any specific species of moth, prohibiting the birds from having any prior preference for any specific patterning. However, examples of edge and corner marginal markings, similar to our treatments, can be found in nocturnal lepidopteran wing patterns. For example, the common peach blossom moth (*Thyatira batis*) from Central Europe (Figure 1a) exhibits disruptive colouration in the form of internal and marginal spots. Cott (1940) identified this species in his early studies of disruptive colouration, as its marginal pink spots contrast with most natural backgrounds. Similar marginal patterning can be seen in the genus *Pseudepimolis* (Figure 1c,d) and in *Oospila albicoma* (Figure S1b). These spot patterns exhibit edge enhancement and high‐contrast boundaries, potentially providing false depth cues which visually split the surface into separate segments (Egan et al. 2016).

The current study targets were modelled for avian vision to best match the bark backgrounds on which they were placed. The dominant trees in the region where the fieldwork was conducted, in southwest Western Australia, are marri (*Corymbia callophylla*) and jarrah ( *Eucalyptus marginata* ). We therefore first measured the colour of the bark of these Eucalypts using photography and image analysis methods. Ten photos were taken using a digital SLR camera (Nikon D7100 with a 60 mm Nikkor lens) of each tree species in Kings Park, Perth, Western Australia. MicaToolbox (Troscianko and Stevens 2015), a plugin for ImageJ software (Schneider et al. 2012) was used to calibrate and linearise these images and model them for bird vision. Multispectral images were created, with three bark regions of interest (ROIs) created for each tree, followed by chart‐based cone catch modelling. We used the blue tit ( *Cyanistes caeruleus* ), a passerine bird, as a reference species to create the visual models. This was due to a lack of knowledge on the spectral sensitivities exhibited by local insectivorous birds.

A batch multispectral image analysis, implemented in MicaToolbox (Troscianko and Stevens 2015), was used to produce cone catches for a bird's short, medium and long wavelength sensitive cones, as well as luminance outputs (double cones), for the ROIs. We did not model cone catches for the UV‐sensitive cones because our camera did not have capability extending into the UV wavelength range. The cone catch values for marri and jarrah bark were similar and were therefore combined to create ‘average Eucalypt bark’ cone catches of 0.140, 0.186, 0.174 and 0.175 for the short, medium and long wavelength and double cones respectively. Six sample paint colour swatches were painted on a 3D‐printed grid and then photographed in situ. These sample colours were linearised and modelled under bird vision using the same chart‐based cone catch modelling as before. We identified the paint colour that most closely matched the average tree bark colour as modelled for bird vision. Colour was the same for all targets and hand painted with two coats (Dulux: Bronze A215; Figure 2). This colour was deemed closest to the average colour of marri (*Corymbia callophylla*) and jarrah trees ( *Eucalyptus marginata* ), on which the treatments were placed. Pin holes were then hand drilled at the top of each target, to facilitate attachment to trees.

### 2.2 Experiment 2: 2D Target Design

We created 2D targets by capturing images of the 3D targets (*n* = three images of each treatment) under natural illumination (25th October in Perth, Western Australia; sun's Azimuth = 75°/Northeast, elevation = 44°). The RAW images were linearised and modelled for bird vision using MicaToolbox (Troscianko and Stevens 2015), as described above (Experiment 1: 3D target design). We then used an iterative printer calibration process (using custom scripts, written by J. Troscianko) that allowed us to print samples of 1026 different colours and to select the one most similar to the average Eucalypt bark modelled for bird vision.

### 2.3 Field Trials

Experiments 1 and 2 were both carried out in spring 2023 and autumn 2024, in south‐west Western Australia (Figure S2). We performed nine blocks for each experiment in Preston National Park (33° 35′ 32.2″ S, 116° 08′ 20.5″ E), near the town of Noggerup, from the 1st to the 9th of November, 2023. Another nine blocks for Experiment 1 and six blocks for Experiment 2 were carried out in nearby Wilga State Forest (33° 37′ 20.1″ S, 116° 12′ 07.9″ E) from the 1st to the 9th of April, 2024. A variety of insectivorous birds were observed to inhabit these forests, including rufous treecreepers ( *Climacteris rufus* ), the western yellow robin ( *Eopsaltria griseogularis* ) and scarlet robins ( *Petroica boodang* ).

Each block was spaced out at least 500 m apart to prevent birds from observing the experiment and identifying human presence as a food source. For both Experiments 1 and 2, each block had 10 targets per treatment, with 60 targets per block. The order of target placement was randomised for each block using a custom‐written R script (R Core Team 2024). Targets were pinned on marri and jarrah trees in approximately 500 m long transects, with trees selected that were at least 3 m apart. All targets were pinned at heights between 1.2 and 1.5 m above ground with a corner pointing up. Tree circumference ranged from 30 to 130 cm. Targets were orientated randomly (in relation to the sun) for each tree. To provide a food source for the birds, the bottom half of a live mealworm, *Tenebrio molitor* , was pinned below each target (exposed tail lengths ~1.5 cm).

The targets were placed out between 10 and 11 am for each block. They were then checked every 3, 6 and 21 h (Experiment 1) or 3, 5 and 19 h (Experiment 2). At each check, we noted signs of predation by free‐living birds (i.e., mealworm missing). If ants were visible near the target, then potential predation by ants was noted. The type/size of ant was noted (e.g., bull ant, medium ant, black ant, small brown ant), as more specific ant identification was not achievable in the field. If the mealworm exoskeleton was present, then potential predation by spiders was noted. We scored ‘1’ for predation by bird and ‘0’ (‘censored’ event for survival analysis; see below) if no predation event by birds had occurred by the last check time (i.e., mealworm present). Targets were considered censored if a non‐avian predation event had likely occurred (i.e., spider/ants).

As the visual metrics of the prey targets will differ, owing to the presence of shadows in prey with disrupted surfaces (King et al. 2023), we photographed targets in situ to determine whether variation in luminance and contrast explained differences in prey survival. To reduce the number of images that were analysed, we photographed targets in situ at the final check time (21 h). For every treatment type that was predated by a bird, we captured an image of the same treatment type (the next that was encountered) that survived, yielding a similar number of images of treatments that had undergone predation or survived. Targets were photographed under natural illumination using a DSLR Nikon D7100 fitted with a 60 mm Nikkor macro lens. Each photograph was taken approximately 50 cm from the tree, with a grey standard (18%) included in each image for subsequent image linearisation and calibration. All remaining pins and targets were removed at the final check.

### 2.4 Statistical Analysis

All statistical analyses were undertaken using RStudio software (ver. 4.4.2) (R Core Team 2024). Cox mixed‐effects models, using the ‘coxme’ package (Therneau 2024a), were used to model the fixed effect of treatment (six levels: I, O, CI, CO, EI and EO) on survival time (in hours). Initially, we conducted separate survival analyses for Experiment 1 (3D targets) and Experiment 2 (2D targets). Survival time was the duration in hours from when the targets were set out to the check time at which a predation event by birds occurred (3, 6 and 21 h in Experiment 1 and 3, 5 and 19 h in Experiment 2). To account for our spatial and temporal sampling design, we entered ‘block’ (number of replicates) and ‘season’ (November/April) as random effects in all models.

We tested for a significant effect of treatment on survival by comparing the full, fitted model to a null model using Log Likelihood Ratio tests (LRTs). We tested the assumptions of the survival models using the ‘survival’ (Therneau 2024b) and ‘survminer’ (Kassambara et al. 2021) packages in R, using Cox proportional hazards models to examine deviance of the residuals and to test for the effect of influential observations. In Experiment 1 (3D targets) there was a total of 1078 target observations, comprising 18 blocks, while in Experiment 2 (2D targets) there was a total of 894 observations over 15 blocks. In our final analysis, we combined the data from Experiments 1 and 2 to determine whether there was a difference in survival probability between 3D and 2D target treatments (*N* = 1972 target observations). For this, we entered the effect of target type (3D or 2D), treatment, and the target type × treatment interaction as fixed factors, and block (33 levels) and season (two levels) as random factors.

### 2.5 Image Analysis

Raw images were converted into calibrated and linearised multispectral images using MicaToolbox (Troscianko and Stevens 2015) in ImageJ (Schneider et al. 2012). We then created a region of interest (ROI) around each target using the polygon tool, taking care to avoid using any images with shadows or strong directional reflectance. We then used the batch multispectral image analysis tool in MicaToolbox (Troscianko and Stevens 2015) to convert the images to a cone catch model (using a chart‐based method, based on the known reflectance values of the Calibrite ColorChecker Passport). We used the bluetit as the observer (without ultraviolet sensitivity: trichromatic vision, because our camera is not modified for UV photography) and average daylight (D65; 400–700 nm; the CIE standard illuminant) as the illumination conditions. This process produced mean and standard deviations for the short, medium and long wavelength sensitive cones, as well as achromatic luminance outputs (double cones) for each ROI. We focussed on the birds' double cones for all subsequent analyses, as achromatic information is regarded as essential for avian visual detection (Osorio and Vorobyev 2005).

We used the R package ‘rstatix’ (Kassambara 2023) in RStudio (R Core Team 2024) to identify and remove seven outliers (> 1.5 IQR from Q1 or Q3) from both the 3D target image dataset (reduced *n* = 238) and the 2D image dataset (reduced *n* = 385 images). We then calculated standardised mean target luminance by dividing each luminance value by the mean. We used the standard deviation of target luminance (hereafter ‘contrast’) as a measure of the internal contrast of each target. As above, block and season were entered as random effects. We entered standardised luminance and contrast as covariates in our analyses to determine whether these visual metrics influenced survival times. We determined the significance of the covariates by comparing each model (e.g., treatment + luminance) to the base model (e.g., treatment only model or null model). To determine whether the targets differed in luminance and contrast, we used ANOVA, followed by Tukey tests with adjustment for multiple comparisons.

## 3 Results

### 3.1 Experiment 1: 3D Targets With Physical Surface Disruption

**FIGURE 3:**
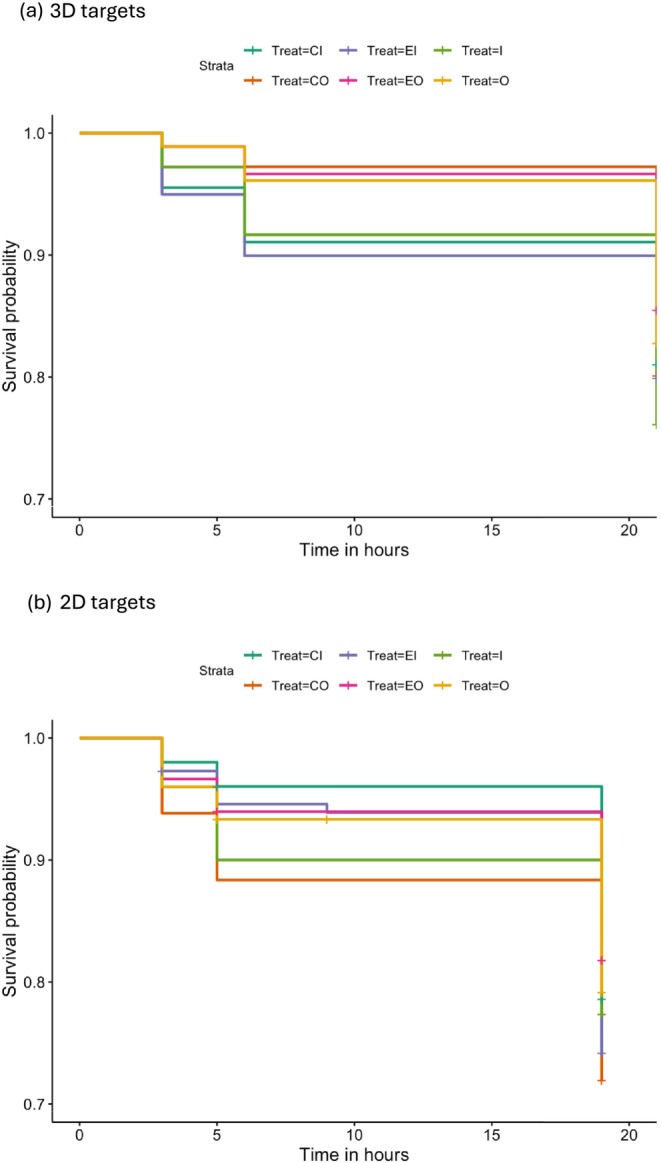
Kaplan–Meier survival curves displaying the variation in survival probability of 3D (a: Experiment 1) or 2D (b: Experiment 2) moth targets with disrupted surfaces (CI, corners in; CO, corners out; EI, edges in; EO, edges out) or continuous surfaces (I, in; O, out) over three check times (3, 6 and 21 h). *N* = 1078 target observations.

Out of the total of 1078 targets placed on trees, there were 206 predation events, or 19.1% probability of predation by free‐living birds over the 21 h observation period. Overall survival was, on average, high during the experiment, ranging from 100% survival at 0 h, approximately 97% at 3 h, 94% at 6 h, and 80% at 21 h. The Cox mixed effects proportional hazards models revealed that there was no effect of treatment on the risk of predation by birds (χ^2^ = 5.42, df = 5, *p* = 0.37; Table 1 and Figure 3a). There was also no difference in risk between the two control targets with undisrupted surfaces (I and O) and the four targets with disrupted surfaces (CI, CO, EI, EO; χ^2^ = 0.44, df = 1, *p* = 0.51).

**TABLE 1:** The results of the Cox mixed‐effects models testing for an effect of treatment (a), treatment and luminance (b) or treatment and contrast (c) on survival time of 3D models.

Treatment	Coef	Exp (Coef)	SE (Coef)	*Z*	*p*
**(a)**					
CO	−0.0474	0.9537	0.2396	−0.20	0.843
EI	0.0794	1.0826	0.2392	0.33	0.740
EO	−0.3326	0.7171	0.2607	−1.28	0.202
I	0.1846	1.2028	0.2300	0.80	0.422
O	−0.1527	0.8584	0.2485	−0.61	0.539
**(b)**					
CO	0.0246	1.0249	0.3134	0.08	0.938
EI	0.0160	1.0161	0.3573	0.04	0.964
EO	−0.0165	0.9836	0.3721	−0.04	0.965
I	0.0343	1.0349	0.3319	0.10	0.918
O	−0.0422	0.9587	0.3257	−0.13	0.897
**(c)**					
CO	−0.0678	0.9345	0.3196e	−0.21	0.832
EI	−0.1884	0.8283	0.3654	−0.52	0.606
EO	−0.2024	0.8168	0.3721	−0.54	0.586
I	0.2193	1.2450	0.3511	0.62	0.532
O	−0.0359	1.0370	0.3274	0.11	0.913

*Note:* Outputs of the models include the coefficient, exponential of the coefficient (HR: hazard ratio), the standard error of the coefficient, Z‐statistic, and *p*‐value. These models account for the random effects of block and season. The CI (‘corners in’) target is the baseline treatment to which the hazard ratios of the other treatments are compared. A hazard ratio of > 1 indicates a higher risk of predation, while a hazard ratio of < 1 indicates a lower risk relative to the baseline. There were 1078 3D target observations and 238 images.

The random effects of block and season accounted for significant variation in our models: 0.37 (SD = 0.61) and 0.13 (SD = 0.36) respectively. We further investigated these effects by entering them (separately) as fixed factors in a Cox proportional hazards model. Blocks 8 and 9 had the lowest survival (Hazard Ratios = 2.19; CI: 1.07–4.49) and 4.50 (CI: 2.28–8.89), while survival was highest in blocks 15 and 18 (HR = 0.25; CI: 0.07–0.91) and 0.17 (CI: 0.04–0.75) (Figure S3a). There was also a significant effect of season, with the relative risk of predation being lower in spring (November) than in autumn (April) (HR = 0.54; CI: 0.41–0.72; Figure S4a). Many of the targets were censored (i.e., taken by non‐bird predator) due to likely ant activity since ants were observed on 443 of the targets placed out, equating to 41% of all 3D targets pinned on trees (Figure S5).

#### 3.1.1 Effect of Image Metrics on 3D Target Survival

Using the reduced dataset incorporating the image data for the final check (21 h), we found that the addition of mean luminance did not improve the fit of the model when compared to a treatment‐only model (χ^2^ = 1.43, df = 1, *p* = 0.23). There was also no effect of luminance alone when compared to the null model (χ^2^ = 1.54, df = 1, *p* = 0.21). The findings were the same for contrast (the standard deviation of target luminance); the addition of contrast did not influence the outcome of a model containing the effect of treatment (χ^2^ = 2.51, df = 1, *p* = 0.11) and there was no effect of contrast alone on the risk of predation by birds (χ^2^ = 1.26, df = 1, *p* = 0.26).

**FIGURE 4:**
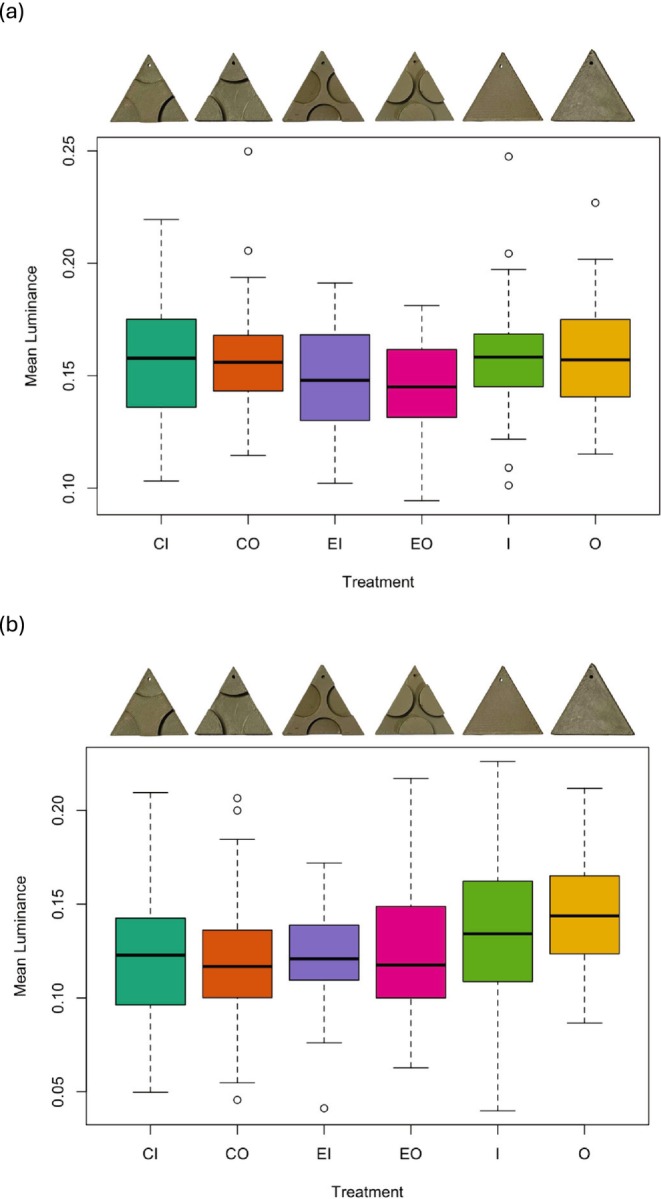
Boxplots displaying the differences in mean luminance amongst the 3D (a) and 2D (b) treatment groups (CI, corners in; CO, corners out; EI, edges in; EO, edges out; I, in; O, out). The sample size consisted of 238 images across the six treatment types. Images of each target type are shown above each boxplot. There is no significant difference among treatments for the 3D targets (a), but for the 2D targets, treatment ‘O’ had higher luminance than all the other treatments (b).

**FIGURE 5:**
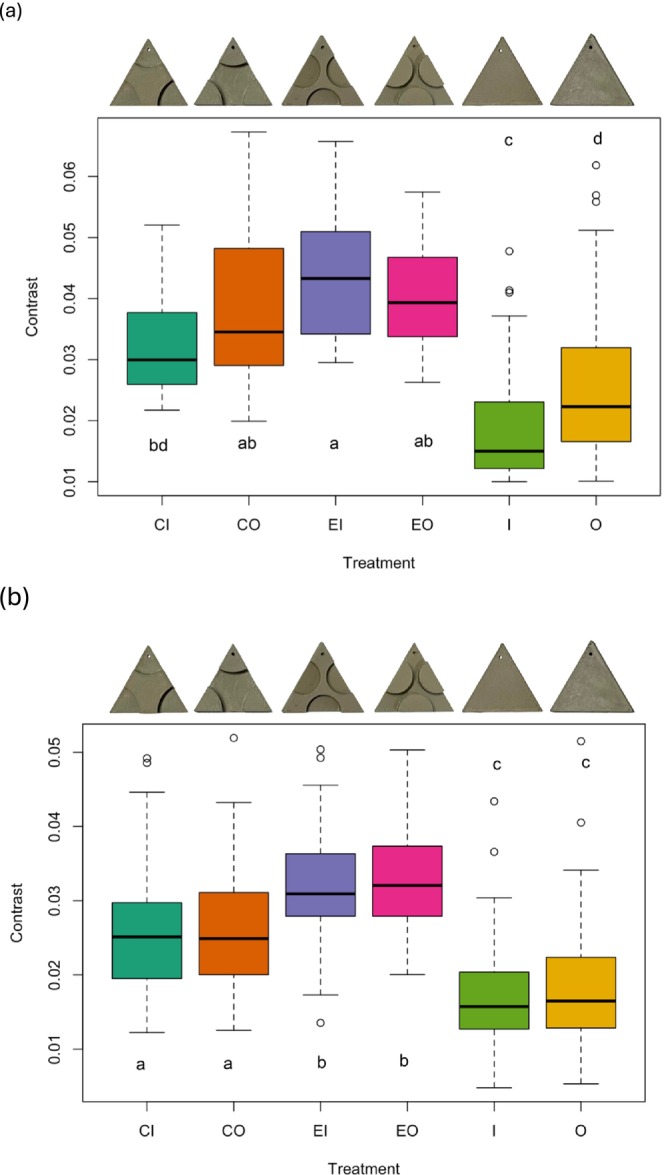
Boxplots displaying the differences in contrast (standard deviation of mean luminance) amongst the 3D (a) and 2D (b) treatment groups (CI, corners in; CO, corners out; EI, edges in; EO, edges out; I, in; O, out). The sample size consisted of 238 images. The letters indicate the significant differences between the treatments (same letters = no significant difference between treatments).

Although target visual metrics did not influence survival, the targets varied in both luminance (*F*_5,232_ = 2.32, *p* = 0.044) and contrast (*F*_5,232_ = 28.53, *p* < 0.001). However, for luminance, none of the post hoc pairwise comparisons were significant after correcting for multiple comparisons (adjusted *p* values all > 0.05; Figure 4a). When considering target contrast, there were significant differences between control targets and all disrupted treatments, except for the O‐CI comparison (Figure 5a).

### 3.2 Experiment 2: 2D Targets With Pictorial Surface Disruption

In Experiment 2, 894 targets were placed on trees and there was a total of 203 predation events, or 22.7% probability of predation by free‐living birds over the 19 h observation period. As in Experiment 1, overall survival was therefore high, ranging from 100% survival at 0 h, 96% at 3 h, 93% at 5 h, and 77% at 19 h.

There was no significant effect of treatment on survival probability (χ^2^ = 5.35, df = 5, *p* = 0.38; Table 2a and Figure 3b). The random effects of block and season explained a large amount of variance in the fit of the model (block: variance = 0.119; season: variance = 0.383). As in Experiment 1, there was significant variation in predation risk explained by both block and season, with survival being relatively low in blocks 10 (HR = 2.81; CI: 1.35–5.8) and 13 (HR = 4.05; CI: 1.96–8.4) (Figure S3b). As in Experiment 1, the relative risk of predation was higher in autumn than in spring (HR = 2.40; CI: 1.8–3.2) (Figure S4b). As in Experiment 1, ants were observed on 381 of the 894 targets that were placed (43% of targets censored due to ants) (Figure S5).

**TABLE 2:** The results of the Cox mixed‐effects survival analysis for 2D targets with pictorial cues (CI = corners in; CO = corners out; EI = edges in; EO = edges out; I = in; O = out), testing for an effect of treatment (a), treatment and luminance (b) and treatment and contrast (c).

Treatment	Coef	Exp (Coef)	SE (Coef)	*Z*	*p*
**(a)**					
CO	0.314	1.368	0.236	1.33	0.184
EI	0.190	1.210	0.240	0.79	0.428
EO	−0.192	0.826	0.261	−0.73	0.464
I	0.092	1.096	0.246	0.37	0.709
O	−0.040	0.961	0.252	−0.16	0.873
**(b)**					
CO	−2.474	0.781	0.289	−0.86	0.392
EI	0.219	1.245	0.268	0.82	0.414
EO	−0.123	0.884	0.303	−0.41	0.684
I	−0.236	0.790	0.304	−0.78	0.437
O	−0.151	0.860	0.301	−0.50	0.615
Luminance	−0.490	0.613	0.368	−1.33	0.183
**(c)**					
CO	−0.225	0.799	0.288	−0.78	0.436
EI	0.284	1.329	0.277	1.03	0.305
EO	−0.059	0.943	0.316	−0.19	0.853
I	−0.374	0.688	0.326	−1.15	0.250
O	−0.311	0.732	0.320	−0.97	0.332
Contrast	−9.740	0.000	12.39	−0.79	0.432

*Note:* The hazard ratio (HR) is given by the exponent of the coefficient (Exp(Coef)) and represents the risk relative to the baseline treatment, which is CI (corners in). A hazard ratio of > 1 indicates a higher risk of predation relative to the baseline, whilst a hazard ratio of < 1 indicates a lower risk relative to the baseline. The random effects of block (15‐levels) and year (2‐levels) were included in the model and total sample size = 894 targets and 385 images.

#### 3.2.1 Effect of Image Metrics on 2D Target Survival

When we re‐ran our analyses on the reduced dataset containing image data (*n* = 385 observations), adding target luminance (χ^2^ = 1.76, df = 1, *p* = 0.18) or target contrast (χ^2^ = 0.63, df = 1, *p* = 0.43) did not change the outcome of the model. There was also no effect of luminance (χ^2^ = 2.31, df = 1, *p* = 0.13) or contrast (χ^2^ = 0.12, df = 1, *p* = 0.73) when these variables were tested alone against the null model. When we examined the visual metrics of the targets, we found that they differed in mean luminance; the ‘O’ target (no pictorial cues) had higher luminance than all other targets except ‘I’ (*F*_5,379_ = 5.68, *p* < 0.001; Tukey test, adjusted *p* < 0.05; Figure 4b). The targets also differed in contrast; all 2D targets with disrupted surfaces had higher contrast than those with non‐disrupted surfaces (*F*_5,379_ = 47.8, *p* < 0.001; Tukey test, adjusted *p* < 0.05; Figure 5b).

### 3.3 Both Experiments Combined: 3D Versus 2D Targets

**FIGURE 6:**
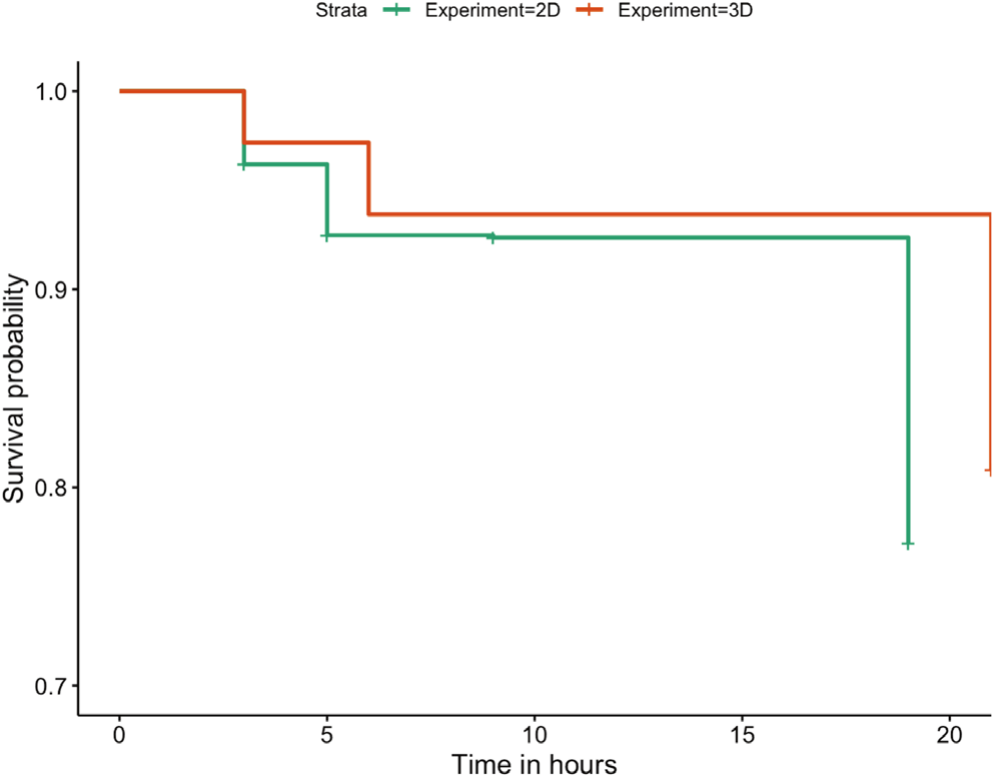
Kaplan–Meier survival curves displaying differences in the survival probability of 3D (red line) and 2D (green line) targets used in Experiments 1 (*N* = 1078 observations) and 2 (*N* = 894 observations) respectively.

When we combined the data from both Experiments 1 and 2, there was no significant interaction between target type (3D or 2D) and treatment. However, there was a significant effect of target type when tested against the null model (χ^2^ = 29.17, df = 1, *p* < 0.001); 3D targets had higher survival than 2D targets (coefficient ± se = −1.48 ± 0.23, HR = 0.229, *z* = −6.5, *p* < 0.001; Figure 6). There was no effect of treatment when compared to the null model (χ^2^ = 8.48, df = 5, *p* = 0.13).

## 4 Discussion

Our study investigated whether the location of surface disruption—at the corners or the edges of a moth‐like target—affected the risk of predation from wild, free‐living birds. Based on our knowledge of human vision (Kristjánsson and Tse 2001), we expected that 3D targets with corner disruption should be harder to recognise and hence should have higher survival than 3D targets with edge disruption. We also anticipated that if pictorial cues produce a percept of depth, then the findings for 2D targets (Experiment 2) should be the same as for 3D targets (Experiment 1). Despite these clear predications, we found no effect of treatment on survival. Instead, survival was strongly influenced by seasonal and spatial factors, suggesting that risk fluctuates according to variation in the local predator community.

The concept of surface disruption has only recently been explored using 3D shapes (King et al. 2023) or objects with holes (Costello et al. 2020), rather than 2D images with potential depth cues (Adams et al. 2019; Egan et al. 2016; Osorio and Srinivasan 1991; Sharman et al. 2018). The former studies (Costello et al. 2020; King et al. 2023) have shown that Lepidoptera‐like targets with disrupted surfaces had a higher probability of survival than controls without disrupted surfaces, supporting the idea that surface disruption may benefit prey by interfering with boundary resolution. However, despite being conducted in the same general study region (southwest Western Australia; King et al. 2023), these results were not replicated in the current study; we found no survival benefit of surface disruption for either 3D or 2D targets.

One possible reason why targets with surface disruption (3D targets) or disruptive colouration (2D targets) did not show higher survival than controls with continuous surfaces is that they were designed to match the average bark background (modelled for chromatic and achromatic bird vision). The targets and controls may therefore have been equally camouflaged (using the mechanism of background matching) with no additional potential benefits provided by surface disruption or disruptive colouration. Indeed, in a similar series of experiments with 2D moth‐like targets, Schaefer and Stobbe (2006) found that cryptic and disruptively coloured moths had similar survival on backgrounds that matched cryptic prey; yet on a mismatched background, edge disruption provided a survival advantage over crypsis. As edge disruption can be effective independent of the background (Cuthill et al. 2005; Schaefer and Stobbe 2006), extending our study to different (i.e., non‐matched) backgrounds may have yielded different results and a survival benefit for prey with surface disruption and/or disruptive colouration.

Another possible reason for the lack of a treatment effect in this study is that the patterns used for disruptive colouration of the 2D targets were images of the 3D targets photographed under natural illumination conditions. Disruptive colouration is most effective if the edge markings are high contrast (Cuthill et al. 2005; Stevens et al. 2006), so it is feasible that these markings, produced by shadows of the 3D target shapes, were not salient enough to interfere with edge detection and boundary resolution. In fact, disruptive patterns often feature patches of colour separated by high contrast lines (known as ‘enhanced edges’) which excite the edge detectors much more strongly than any shadows created by the animal's natural boundaries (Osorio and Srinivasan 1991). This may explain why studies that have created disruptive patterning using enhanced edges have reported survival benefits of these markings (Adams et al. 2019; Egan et al. 2016; King et al. 2023; Sharman and Lovell 2019), while those that have used images of 3D surfaces with shadows have not (Kelley et al. 2023; current study).

The effects of season and temperature may explain the low predation rates in this study compared to King et al. (2023). King et al. (2023) conducted their experiment during April–May (Autumn) and found that survival probability ranged from 38% to 60% at the final 19 h check time. While the highest bird predation in our experiment was in early April, survival rates remained higher overall (77%–80% survival at the final check time), suggesting that predator activity during our experiment was low. This seasonal variation in predation coincides with the observed foraging preferences of native rufous treecreepers ( *Climacteris rufa* ), which we observed in situ in autumn, and which tend to forage on tree bark in autumn but shift to ground foraging in winter (Luck 2001). Additionally, Luck (2001) found that these birds' food provisioning rate increases in spring, with the number of nestlings. Based on these findings, future experiments could benefit from trialling fieldwork in the early spring months (September and October) to coincide with the breeding season and higher bird foraging activity. It is important to note that we do not have any direct evidence of avian predation, despite trialling the use of modelling clay instead of meal worms (they were not attacked by birds) and placing camera traps alongside a subset of targets (the frame rate was not sufficient to capture avian predators in action). As in similar studies using this method, it is therefore unclear whether our low predation rates are because avian predators do not detect the targets or whether it is because they detect them and choose not to approach them. This is a challenge for future studies and critical to our understanding of camouflage, where some strategies are based on avoiding detection while others are based on impeding recognition (Merilaita et al. 2017).

Neophobia in wild birds may have contributed to the lower levels of predation in these experiments (i.e., the current study; King et al. 2023) compared to European studies, which tend to report higher target predation rates (~20%–40% survival), albeit over longer experimental periods (up to 54 h; Costello et al. 2020; Cuthill et al. 2005; Schaefer and Stobbe 2006). Neophobia is the phenomenon of wild birds avoiding novel, unfamiliar objects or situations, which may represent a threat to their survival (Greenberg and Mettke‐Hofmann 2001). Here, the 3D targets were a novel stimulus, as bark‐resting moths are generally flat and would therefore have closer resemblance to the 2D targets. This may account for the increased survival probability of 3D targets over 2D targets in both this study and in King et al. (2023). This, and previous experiments with 3D moth‐like prey (King et al. 2023) have been conducted in relatively remote forests with minimal human activity, so disturbance by the experimenters may explain the low predation rates. Since reduced neophobia has been observed in urban‐nesting birds (Heales et al. 2024), repeating this experiment in an urban environment may offer higher rates of predation. This may include priming the birds on control treatments, to recognise them as a food source before exposing them to an array of surface‐disrupted treatments.

During the experiment, we were careful to record any evidence of ants on or around the tree that the target was placed on, so that we did not overestimate predation rates. This is because ants with large mandibles, such as bull ants (*Myrmecia* spp.), were observed (RM, pers. obs.) to remove mealworms in under an hour, whilst large numbers of smaller ants were observed removing mealworms overnight (captured on camera trap footage). Ant activity is the reason why we did not measure survival over longer durations or space out the target checks. Examining temperature data during our study period, it was evident that predation by birds increased with cooler temperatures, while ant interference was increased at warmer temperatures (Figure S5). Future experiments should consider the activity of both birds and ants. For example, it is known that the foraging activity of rufous treecreepers is high in the early morning and at cooler temperatures (Luck 2001), while in ants, activity levels for multiple species are positively correlated with temperature (Majer and Koch 1982). Ant interference and warmer temperatures are just some of the varying challenges posed by the Australian ecosystem for field experiments on avian predation.

In this experiment, the location of targets on trees was random, and all targets had a consistent vertical positioning (i.e., one corner orientated upright), which is unlikely to reflect the preferred positioning of a moth resting in the wild. Indeed, moths may choose to dynamically align themselves with particular bark furrows and textures for optimal camouflage and breaking up of edges (Kang et al. 2012). Such orientation behaviours may function to defeat biases in predators' visual processing. For example, in human visual systems, more cells are responsive to the horizontal and vertical orientations of a stimulus than to oblique orientations, known as ‘orientation tuning’ (Hansen and Essock 2004). A visual preference for vertical or horizontal lines over oblique ones, the ‘oblique effect’, has also been observed in other primates (Kennedy et al. 1985), cats (Li et al. 2003) and birds (pigeons; Donis 1999). In the case of real moths, the combination of wing patterning and orientation relative to the background colouration will have an important effect on detectability (Kang et al. 2012).

The study of animal camouflage has developed rapidly as an interdisciplinary topic, providing insights beyond evolutionary biology into visual perception in other species (Cuthill 2019). Although we found no evidence that the location of surface disruption increases camouflage efficacy, we consider it important to publish null findings so that others can test the underlying concepts using a different experimental framework. Indeed, we hope that our work will inspire others to look to our knowledge of human visual perception to generate and test new ideas about how camouflage works for non‐human animals.

## Author Contributions

**Ruby McLellan:** data curation (equal), formal analysis (equal), investigation (equal), methodology (equal), writing – original draft (equal), writing – review and editing (equal). **Vanessa K. Bowden:** conceptualization (equal), investigation (equal), methodology (equal), supervision (equal), writing – review and editing (equal). **Troy A. W. Visser:** conceptualization (equal), investigation (equal), methodology (equal), supervision (equal), writing – review and editing (equal). **Jennifer L. Kelley:** conceptualization (equal), data curation (equal), formal analysis (equal), funding acquisition (lead), investigation (equal), methodology (equal), project administration (equal), supervision (equal), writing – original draft (equal), writing – review and editing (equal).

## Conflicts of Interest

The authors declare no conflicts of interest.

## Supporting information


**Data S1:** ece372052‐sup‐0001‐TableS1‐FigureS1‐S5.docx.

## Data Availability

Code and data supporting the analyses are available in Dryad at https://doi.org/10.5061/dryad.v41ns1s83.
