# Supplementary material for: Camouflage Using Surface Disruption: The Importance of Corners Versus Edges
Source: Ecol Evol. 2025 Aug 21;15(8):e72052. doi: 10.1002/ece3.72052 (PMC12370832; doi:10.1002/ece3.72052)
Supplement: Supplementary file 1 — Data S1: ece372052‐sup‐0001‐TableS1‐FigureS1‐S5.docx. [file ECE3-15-e72052-s001.docx]

**Supplementary Material for McLellan et al: Camouflage using surface disruption: the importance of corners versus edges.**

Table S1. The calculations for the target design of (A) edge disrupted treatments (EI and EO), and (B) corner disrupted treatments (CI and CO). The ‘Area_△b centre_’ refers to the area of the remaining connected triangle surface and the ‘Area_△b round_’ refers to the area of the disconnected edge/corner component. The collective surface area of the disconnected components was made to be equal to the remaining surface area. ‘b’ refers to the target edge lengths (4cm), ‘h’ refers to the target height (3.464cm), and ‘θ’ refers to the angle of each corner (60°). For (A) ‘r’ refers to the radius of a disconnected edge component (0.857cm), for (B) ‘r’ refers to the length of a disconnected corner component (1.485cm).

| (A) Calculations for edge disrupted treatments (EI and EO) | (B) Calculations for corner disrupted treatments (CI and CO) |
| --- | --- |
| $\boldsymbol{Equilateral} \boldsymbol{Triangle} \boldsymbol{Properties}$  ${Area}_{\Delta}=\frac{1}{2}bh$  $\theta_{\Delta equilateral}=60^{\circ}$  $h_{\Delta equilateral}=\frac{\sqrt{3}}{2}b$  ${Area}_{\Delta equilateral}=\frac{1}{2}bh=\frac{1}{2}b\left( \frac{\sqrt{3}}{2}b \right)=\frac{\sqrt{3}}{4}b^{2}$  $\boldsymbol{Calculating} \boldsymbol{Area}_{\Delta\boldsymbol{a} \boldsymbol{round}}$  ${Area}_{Semi Circle}=\frac{1}{2}\pi r^{2}$  ${Area}_{\Delta a round}=3*{Area}_{Semi Circle}=3*\left( \frac{1}{2}\pi r^{2} \right)=\frac{3}{2}\pi r^{2}$  $\boldsymbol{Calculating} \boldsymbol{Area}_{\Delta\boldsymbol{a} \boldsymbol{centre}}$  ${{Area}_{\Delta a centre}=Area}_{\Delta a round}$  $\boldsymbol{Calculating} \boldsymbol{Area}_{\boldsymbol{total}}$  ${{Area}_{total}=Area}_{\Delta a round}+{Area}_{\Delta a centre}$  ${{Area}_{total}=Area}_{\Delta a round}+{Area}_{\Delta a round}$  ${Area}_{total}=2*{Area}_{\Delta a round}$  ${Area}_{total}=2*\left( \frac{3}{2}\pi r^{2} \right)$  ${Area}_{total}=3\pi r^{2}$  ${Area}_{total}=$ ${Area}_{\Delta equilateral}$  ${Area}_{total}= \frac{\sqrt{3}}{4}b^{2}$  $\therefore{Area}_{\Delta equilateral}=2*{Area}_{\Delta a round}$  $\boldsymbol{Calculating} \boldsymbol{r} \boldsymbol{in} \boldsymbol{terms} \boldsymbol{of} \boldsymbol{b}$  $\frac{\sqrt{3}}{4}b^{2}=3\pi r^{2}$  $\frac{1}{3\pi}*\frac{\sqrt{3}}{4}b^{2}=r^{2}$  $\frac{1}{4\sqrt{3}\pi}b^{2}=r^{2}$  $\sqrt{\frac{1}{4\sqrt{3}\pi}}*\sqrt{b^{2}}=\sqrt{r^{2}}$  $r=\frac{1}{2*3^{0.25}*\sqrt{\pi}}*b$  $r=0.2143457 b$  $\boldsymbol{Assuming} \boldsymbol{b}=\mathbf{4}\boldsymbol{cm}, \boldsymbol{we} \boldsymbol{can} \boldsymbol{calculate} \boldsymbol{the} \boldsymbol{following};$  $r=0.2143457*4=0.857cm$  $h_{\Delta equilateral}=\frac{\sqrt{3}}{2}4=3.644cm$  ${Area}_{total}= \frac{\sqrt{3}}{4}4^{2}=6.928cm^{2}$  ${Area}_{Semi Circle}=\frac{1}{2}\pi{0.857}^{2}=1.155cm^{2}$  ${Area}_{\Delta a centre}=3*{Area}_{Semi Circle}=3*1.155cm^{2}=3.464cm^{2}$  $\boldsymbol{Checking} \boldsymbol{Values}$  ${Area}_{Semi Circle}=\frac{1}{6}{Area}_{total}=\frac{1}{6}*6.928cm^{2}=1.155cm^{2}$  ${Area}_{\Delta a centre}={\frac{1}{2}Area}_{total}=\frac{1}{2}6.928cm^{2}=3.464cm^{2}$ | $\boldsymbol{Equilateral} \boldsymbol{Triangle} \boldsymbol{Properties}$  ${Area}_{\Delta}=\frac{1}{2}bh$  $\theta_{\Delta equilateral}=60^{\circ}$  $h_{\Delta equilateral}=\frac{\sqrt{3}}{2}b$  ${Area}_{\Delta equilateral}=\frac{1}{2}bh=\frac{1}{2}b\left( \frac{\sqrt{3}}{2}b \right)=\frac{\sqrt{3}}{4}b^{2}$  $\boldsymbol{Calculating} \boldsymbol{Area}_{\Delta\boldsymbol{b} \boldsymbol{round}}$  ${Area}_{Sector}=\frac{\theta}{360}\pi r^{2}=\frac{60}{360}\pi r^{2}=\frac{1}{6}\pi r^{2}$  ${Area}_{\Delta b round}=3*{Area}_{Sector}=3*\left( \frac{1}{6}\pi r^{2} \right)=\frac{3}{6}\pi r^{2}=\frac{1}{2}\pi r^{2}$  ${\boldsymbol{Calculating} \boldsymbol{Area}}_{\Delta\boldsymbol{b} \boldsymbol{centre}}$  ${{Area}_{\Delta b centre}=Area}_{\Delta b round}$  ${\boldsymbol{Calculating} \boldsymbol{Area}}_{\boldsymbol{total}}$  ${{Area}_{total}=Area}_{\Delta b round}+{Area}_{\Delta b centre}$  ${{Area}_{total}=Area}_{\Delta b round}+{Area}_{\Delta b round}$  ${Area}_{total}=2*{Area}_{\Delta b round}$  ${Area}_{total}=2*\left( \frac{1}{2}\pi r^{2} \right)$  ${Area}_{total}=\pi r^{2}$  ${Area}_{total}=$ ${Area}_{\Delta equilateral}$  ${Area}_{total}= \frac{\sqrt{3}}{4}b^{2}$  $\boldsymbol{Calculating} \boldsymbol{r} \boldsymbol{in} \boldsymbol{terms} \boldsymbol{of} \boldsymbol{b}$  $\frac{\sqrt{3}}{4}b^{2}=\pi r^{2}$  $\frac{1}{\pi}*\frac{\sqrt{3}}{4}b^{2}=r^{2}$  $\sqrt{\frac{\sqrt{3}}{4\pi}}*\sqrt{b^{2}}=\sqrt{r^{2}}$  $r=\left( \frac{3^{0.25}}{2\sqrt{\pi}} \right)b$  $r=0.3712576 b$  $\boldsymbol{Assuming} \boldsymbol{b}=\mathbf{4}\boldsymbol{cm}, \boldsymbol{we} \boldsymbol{can} \boldsymbol{calculate} \boldsymbol{the} \boldsymbol{following};$  $r=0.3712576 *4=1.485cm$  $h_{\Delta equilateral}=\frac{\sqrt{3}}{2}4=3.644cm$  ${Area}_{total}= \frac{\sqrt{3}}{4}4^{2}=6.928cm^{2}$  ${Area}_{Sector}=\frac{1}{6}\pi{1.485}^{2}=1.155cm^{2}$  ${Area}_{\Delta a centre}=3*{Area}_{Sector}=3*1.155cm^{2}=3.464cm^{2}$  $\boldsymbol{Checking} \boldsymbol{Values}$  ${Area}_{Semi Circle}=\frac{1}{6}{Area}_{total}=\frac{1}{6}*6.928cm^{2}=1.155cm^{2}$  ${Area}_{\Delta a centre}={\frac{1}{2}Area}_{total}=\frac{1}{2}6.928cm^{2}=3.464cm^{2}$ |

Figure S1. A graphic demonstrating the equilateral triangle target design and dimensions. This visualises the area of each disconnected corner/edge component as being equal to the remaining triangle surface area for all surface disrupted treatments.


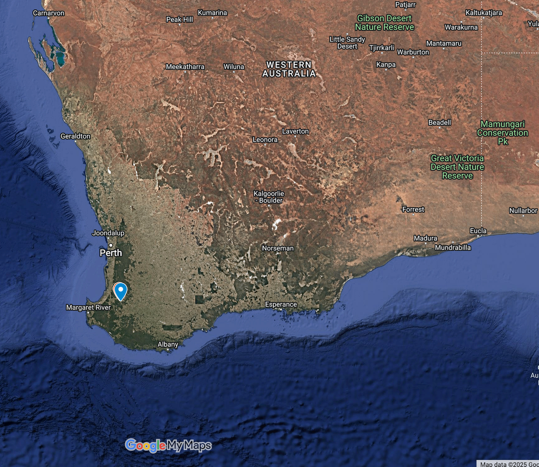

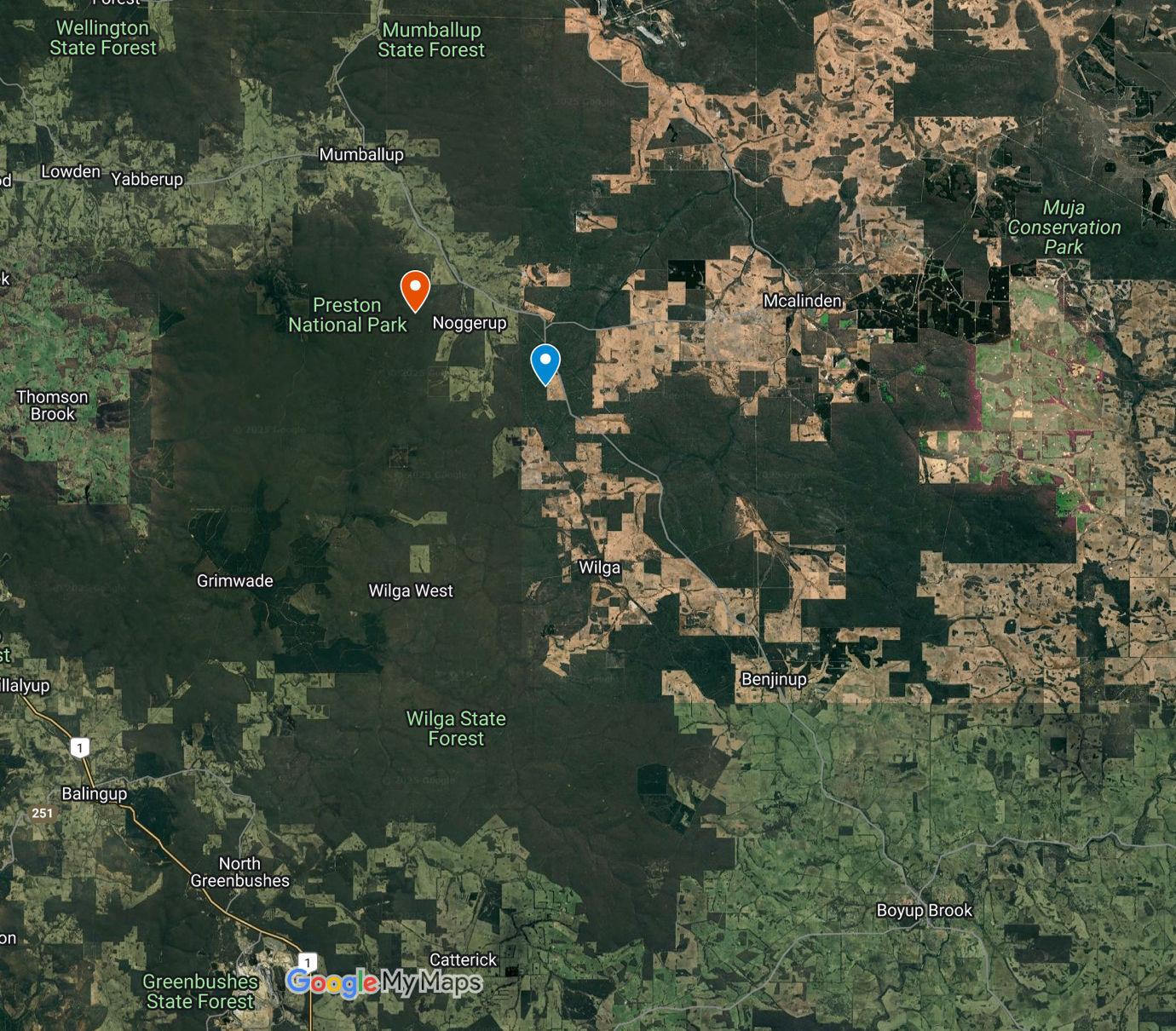


Figure S2. Google My Maps images of the region of southwest Western Australia (inset) where the study was conducted. Experiments 1 and 2 were conducted in Preston National Park (red: 33° 35' 32.2" S, 116° 08' 20.5" E, near the town of Noggerup and Wilga State Forest (blue: 33° 37' 20.1" S, 116° 12' 07.9" E) in austral spring 2023 and autumn 2024.

(a)


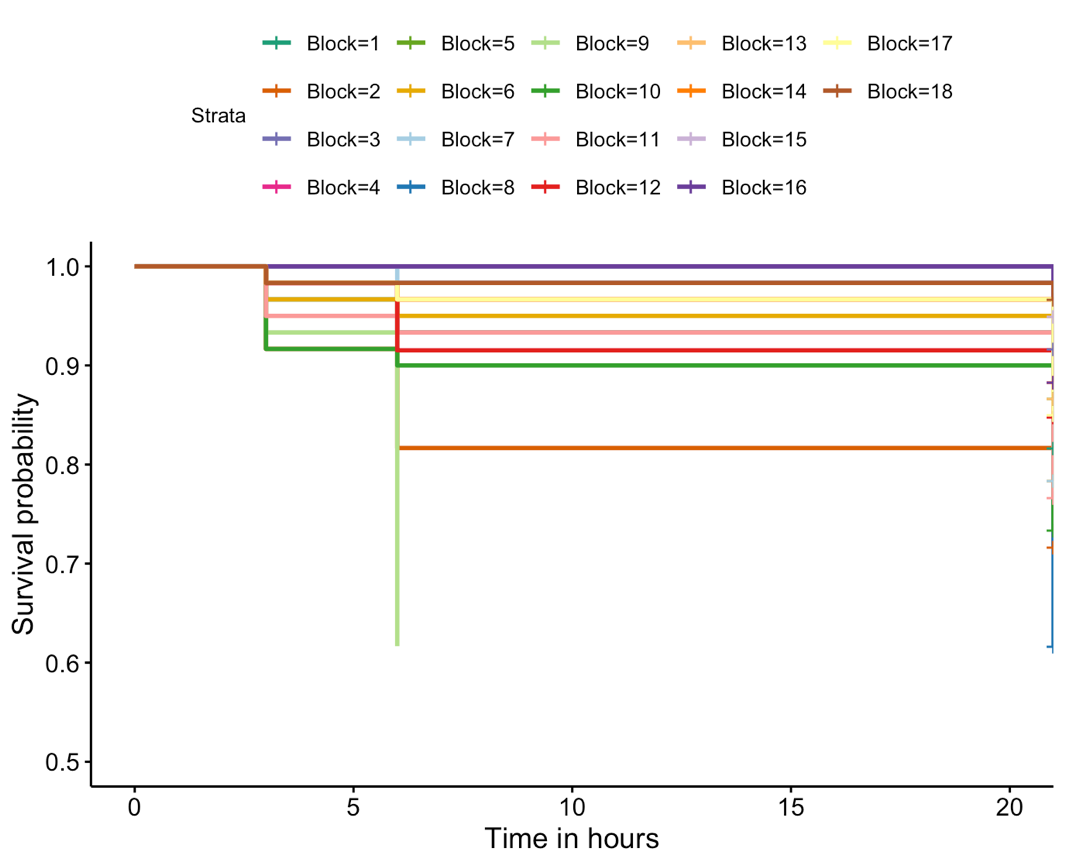


(b)


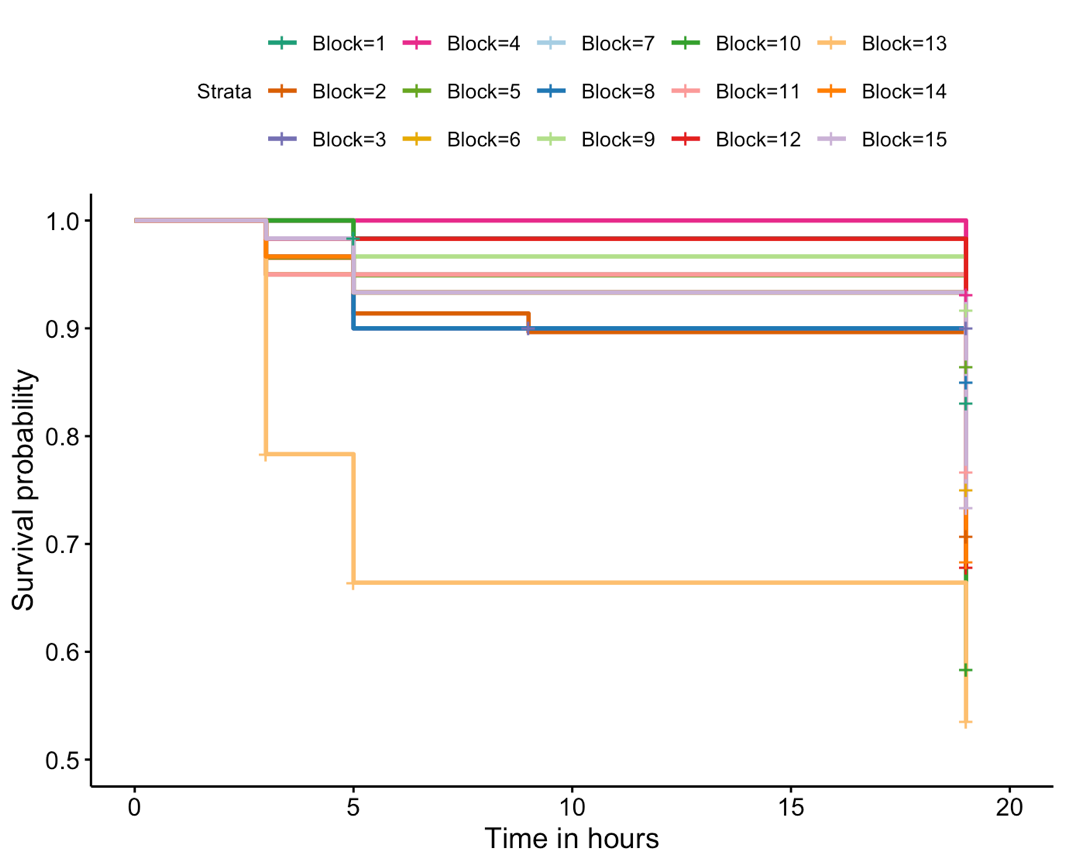


Figure S3. Kaplan-Meier survival curves showing the variation in survival probability of all 3D targets (a; Experiment 1, n = 1078) and 2D targets (b; Experiment 2, n = 894) over 18 (a) and 15 (b) experimental blocks. This occurred over three check times (a: 3, 6 and 21 hours; b: 3, 5 and 19 hours). Blocks 1-9 were undertaken in early April 2024. Blocks 10-18 took place in early November 2023.

(a)


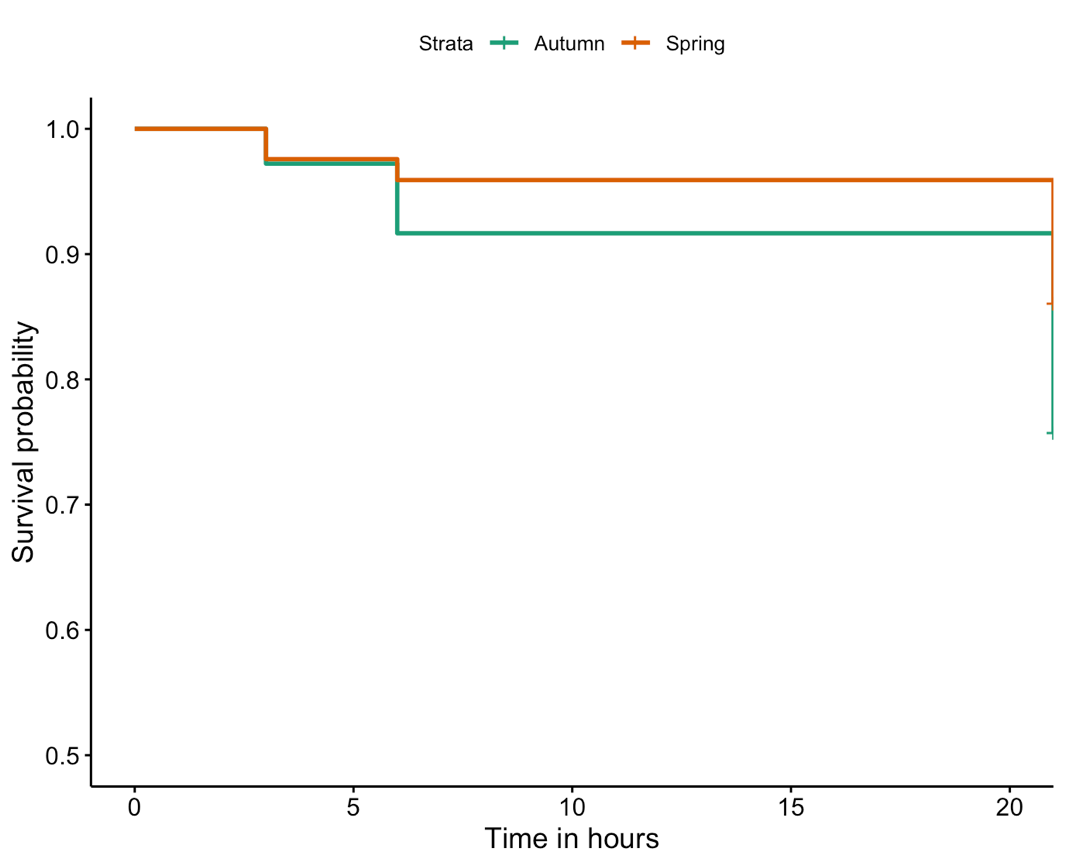


(b)


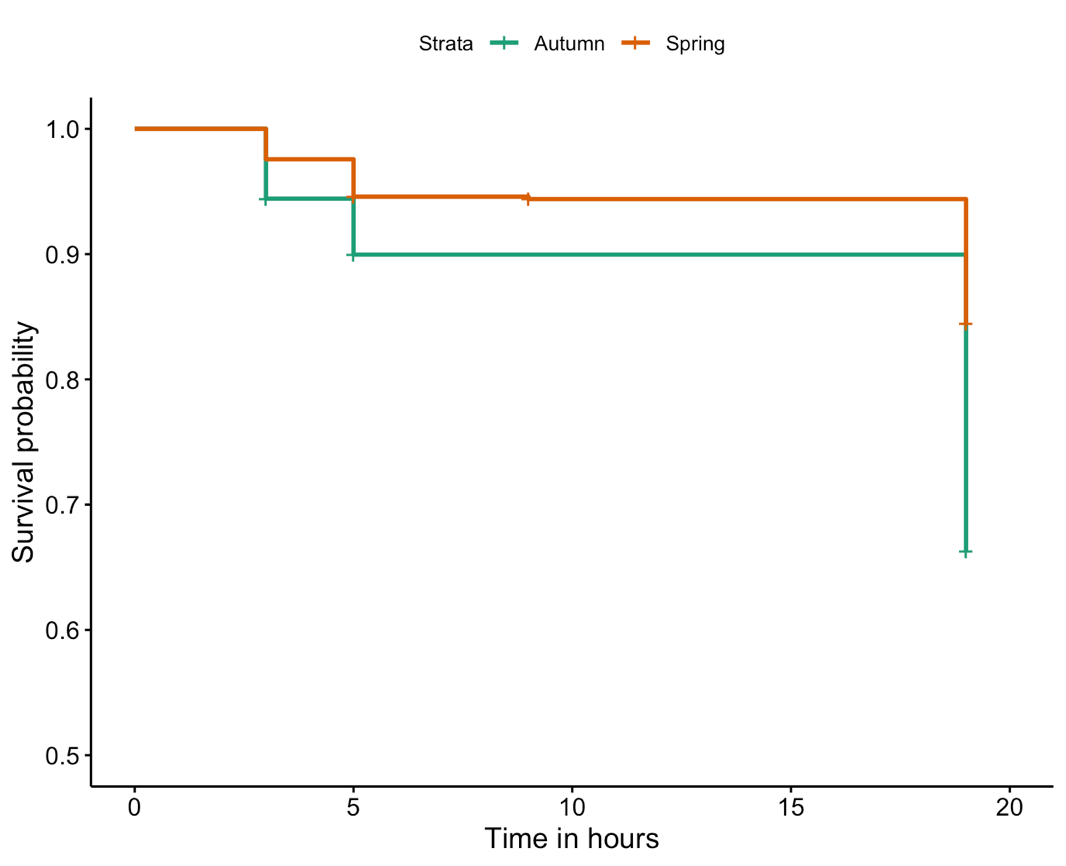


Figure S4. Kaplan-Meier survival curves showing the survival probability of all 3D (a; Experiment 1, n = 1078) and 2D targets (b; Experiment 2, n = 894) targets (n = 894) in spring (blue line, November 2023) and autumn (red line, April 2024). Targets were checked for survival at three check times (a: 3, 6 and 21 hours; b: 3, 5 and 19 hours).

Figure S5. Graph demonstrating the ant interference (red) and bird predation events (count; in green) for each block, including the maximum daily temperature (°C) (yellow). The ant interference count is the number of targets within each block that had ant presence. Data are for Experiment 1; blocks 1-9 were undertaken in early April 2024, in Wilga, blocks 10-18 took place in early November 2023, in Noggerup, Western Australia. Data on daily maximum temperatures were obtained from the Bureau of Meteorology, from the nearest open weather stations for each location (Australian Bureau of Meterology, 2024).

**References**

Australian Bureau of Meterology. (2024). www.bom.gov.au
